# Ectopic endometrial stromal cells‐derived exosomes accelerate endometriosis progression by delivering PDLIM3 to induce macrophage M2 polarization

**DOI:** 10.1002/ccs3.70122

**Published:** 2026-10-23

**Authors:** Chi Chi, Jie Chen, Shuzhen Xiang, Bing Han, Weizeyu Liu, Yi Zhang, Jie Zhang, Min Li

**Affiliations:** ^1^ Department of Obstetrics and Gynecology The First Affiliated Hospital of Soochow University Suzhou Jiangsu China

**Keywords:** endometriosis, exosomes, M2 polarization, NAA40, PDLIM3

## Abstract

Endometriosis (Ems) is a benign disease characterized by invasion and migration. Abnormally expressed mRNAs regulate its progression. In this study, we analyzed GEO datasets and identified significant upregulation of PDLIM3 in ectopic endometrial tissues. Our aim was to explore the role of PDLIM3 and its regulatory mechanisms in Ems progression. Using bioinformatics, we analyzed differentially expressed genes and treated endometrial stromal cells (ESCs) with modified PDLIM3 and PRMT1 expression vectors to assess their impact on Ems. We measured macrophage polarization markers and the secreted cytokines as well as the percentage of CD206‐positive macrophages to evaluate the PDLIM3/NAA40/PRMT1 axis's influence on macrophage polarization. Also, we tested the impact of THP‐1‐derived macrophages (THP‐1‐DM) under different conditions on ESCs. An animal model was developed to study the effects of PDLIM3 carried by exosomes from ectopic ESCs (ecESCs‐Exos) on macrophage M2 polarization and Ems progression. M2 macrophages enhance ecESCs proliferation and migration, with PDLIM3 delivered by exosomes from ecESCs inducing M2 macrophage polarization. Mechanistically, PDLIM3 binds to NAA40 to suppress its ubiquitination and thus stabilize its protein. Then, the enhanced NAA40 binds to PRMT1 promoter and promotes asymmetric dimethylation of H4 histone to activate PRMT1, hence driving M2 macrophage polarization. In turn, in vitro data showed that ecESCs‐Exos‐induced M2 macrophages via PDLIM3/PRMT1 axis further accelerates ESCs proliferation and migration. Moreover, in vivo data demonstrate that exosomal PDLIM3 accelerates Ems progression via contributing to macrophage M2 polarization. Exosomal PDLIM3 from ecESCs contributes to Ems progression by inducing M2 macrophage polarization. The findings provide new clues regarding the progression of Ems, which might be beneficial for developing new therapeutic targets for Ems patients.

## Graphical abstract

ecNSCs‐secreted exosomal PDLIM3 stabilizes NAA40 to epigenetically upregulate PRMT1, driving macrophage M2 polarization.

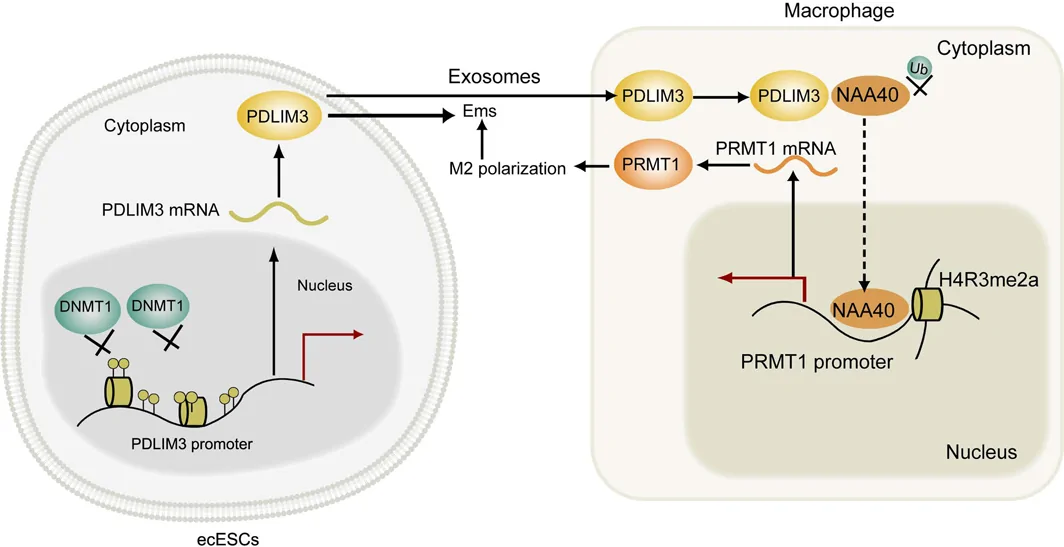

## 1 INTRODUCTION

Endometriosis (Ems) is a gynecological disease in which active endometrial stromal cells (ESCs) are implanted outside the uterine cavity.^1^ Its pathological features were ectopic endometrial periodic bleeding and tissue fibrosis.^2^ Ems has a high incidence and can cause infertility, causing serious health problems for patients.^3^, ^4^ Although multifactorial etiologies in Ems have been explored in past few decades,^5^, ^6^, ^7^, ^8^, ^9^ the pathogenesis of Ems is still unclear. Here, we explore the molecular mechanisms that influence the progression of Ems with significant implications for improving the diagnosis and treatment of the disease.

PDZ and LIM domain 3 (PDLIM3) encodes on chromosome 4q35.1.^10^, ^11^ As a component of the Z line of mature muscle fibers,^12^ its high expression has been detected in cardiac, smooth, and skeletal muscle. PDLIM3 plays critical role in diversified biological activities, including differentiation, proliferation, and signal transduction; moreover, it can affect muscle stability and cytoskeletal structural integration.^13^ Previous reports have revealed the role of PDLIM3 in several human diseases such as muscular dystrophy DCM and human cancers.^14^, ^15^, ^16^, ^17^ However, there is no study reporting the role of PDLIM3 in Ems. This study focused on the functions of PDLIM3 in ESCs proliferation and migration.

Recently, exosomes are reported in Ems due to its regulatory effect on ESCs proliferation and migration.^18^, ^19^, ^20^ A previous study revealed that exosomes derived from Ems mouse model induced macrophage M2 polarization.^21^ More recent studies have also suggested that PDLIM3 has been reported as a potential biomarker of immune infiltration in patients with Ems and is associated with macrophage M2 polarization.^22^ Hence, our study tried to explore whether ecESCs delivers PDLIM3 to induce macrophage M2 polarization through secreting exosomes.

In summary, this study aims to explore the specific functions and regulatory mechanisms of ectopic ESCs‐derived exosomal PDLIM3 in Ems progression and macrophage polarization.

## 2 MATERIAL AND METHODS

### 2.1 Isolation of human ectopic ESCs (ecESCs), normal ESCs (nESCs) and primary human monocyte‐derived macrophages (hMDM)

The approval to this study was obtained from the Institutional Review Board of the First Affiliated Hospital of Soochow University. None of the patients enrolled in this study was undergoing hormonal treatment before surgery. Primary human nESCs were obtained from normally cycling premenopausal women with uterine fibroids through hysterectomy. Human ecESCs were obtained from women with Ems through ovarian cystectomy or oophorectomy. Ovarian endometrioma or Ems was diagnosed and confirmed through histological examination. Isolation and culture of ecESCs and nESCs were conducted as previously described.^23^, ^24^ After isolation and purification, nESCs and ecESCs were cultured in DMEM/F‐12 containing 1% antibiotic–antimycotic solution (Gibco) and maintained at 37°C.

To acquire primary hMDM, mononucleated cells were first isolated from human blood sample of healthy donors via Ficoll Hypaque (CMSMSL01, Eurobio Scientific). Then, monocytes were selected by anti‐CD14 trageting and then maintained in RPMI 1640 medium containing glutamax‐I (Life Technologies, 61870044) and 10% FBS. To obtain hMDM, monocytes were subjected to 50 ng/mL CSF‐1 (130–096‐493, Miltenyi Biotec) to induce macrophage differentiation.

### 2.2 RNA extraction and RT‐qPCR

Using Trizol reagent to isolate total RNA from indicated cells. Reverse transcription of the total RNA was completed by using Bio Script reverse transcriptase (Bioline) with oligo dT primers. qRT‐PCR was carried out using the SYBER Green PCR Master Mix (Applied Biosystems) in the 7900 HT Fast Real Time PCR System (Applied Biosystems). After calculation the relative mRNA expression using the 2^−ΔΔCt^ method,^25^ results were normalizing to GAPDH and shown as mean ± standard derivation (SD).

### 2.3 Western blot

Total protein isolated using RIPA buffer was quantified with a bicinchoninic acid kit (Thermo Scientific, Rockford, IL, USA). Samples were then subjected to separation on sodium dodecyl sulfate‐polyacrylamide gel electrophoresis. After being transferred to a poly (vinylidene fluoride) (PVDF) membrane, samples were incubated with primary antibodies of PDLIM3, CD163, ARG1, MRC1 (also called CD206), CD81, TSG101, calnexin, NAA40, N‐acH4, H4R3me1, H4R3me2a, H4R3me2s and β‐actin at 4°C overnight. After washing, the membranes were further incubated by the secondary antibody. A Tanon‐5200 Image Analyzer (Tanon, Shanghai, China) was used to visualize bands carrying protein blots.

The protein levels of PDLIM3 in THP‐1‐DM cells after treating with Cycloheximide (CHX), MG132 or Bafilomycin A1 (BafA1) for 0, 6, 12 h were also detected by western blot, with band quantification via Image‐Pro Plus 6.0. Besides, the levels of HA‐NAA40 and Flag‐PDLIM3 in THP‐1‐DM cells after being transfected with different dose of plasmids covering HA‐NAA40 or Flag‐PDLIM3 were also evaluated by western blot and then quantified by Image‐Pro Plus 6.0.

### 2.4 Cell transfection

Short‐hairpin RNAs (shRNAs) directed against PDLIM3 (sh‐PDLIM3‐1, sh‐PDLIM3‐2), NAA40 (sh‐NAA40‐1, sh‐NAA40‐2) and their negative control shRNA (sh‐NC) were obtained from GenePharma for knockdown of PDLIM3 or NAA40. To overexpress DNMT1 or PRMT1, the full length of them was separately cloned into pcDNA3.1 vector (GenePharma). The empty pcDNA3.1 vector was used as the control for overexpression vectors. Transfections were carried out using Lipofectamine 2000 (Invitrogen).

### 2.5 CCK‐8 assay

The viability of nESCs or ecESCs was assessed through Cell Counting Kit‐8 assay kit (Beyotime). Briefly, cells were seeded into 96‐well plates, each well was added with 100 μL complete medium for culturing 3000 cells. Next, cells were incubated with 10 μL of CCK‐8 reagent at 37°C for 2 h. Finally, a microplate reader (Thermo Fisher Scientific) was used for the absorbance measurement at 450 nm.

### 2.6 EdU assay

Indicated nESCs or ecESCs were seeded in a 24‐well culture plate at a rate of 2 × 10^5^ cells/well and incubated for 24 h. The EdU analysis kit (Beyotime Biotechnology) was applied for the analysis and evaluation of cell proliferation. The results were observed and analyzed with a Leica fluorescence microscope.

### 2.7 Transwell assay

After indicated treatments, cells were seeded on the upper chamber of a 24‐well plate containing serum‐free medium. The lower chamber was filled with medium containing 10% FBS. Twenty‐eight hours after incubation, discarding the unmigrated cells, fixing the migrated cells, and the dying the migrated cells with crystal violet (Beyotime Biotechnology). Finally, the stained cells were observed and captured using an Olympus microscope (Olympus) at 5 random fields.

### 2.8 Exosome isolation and determination

Exosomes were extracted from ecESCs by ultracentrifugation method as mentioned previously.^26^

The morphology of exosomes was observed by a transmission electron microscopy (JEM‐1400) at 80–120 kV voltage. The diameter and size distribution of exosomes was detected by Nanoparticle tracking analysis (NTA).

Exosomes derived from ecESCs were labeled with PKH26 kit (PKH26 Red Fluorescent Cell Linker Kit, Sigma). Briefly, ecESCs were fixed by 4% paraformaldehyde, stained with DAPI, and washed with PBS for three times. Using a Carl Zeiss LSM710 laser scanning confocal microscope to observe the PKH26 fluorescence signal.

### 2.9 Flow cytometry

THP‐1‐DM cells were blocked with anti‐immunoglobulin antibody, collected with trypsin, and rinsed with PBS. THP‐1‐DM cells were stained by Phycoerythrin (PE)‐Cluster of Differentiation 206 (CD206) at 4°C under dark conditions for 30 min. The percentage of CD206‐positive macrophages in THP‐1‐DM cells were detected by FACSAria (BD Biosciences) using FACSDiva software (BD Biosciences, USA).

### 2.10 Co‐IP and mass spectrometry analysis

To detect PDLIM3‐interacting proteins, cells were lysed in NP‐40 lysis buffer (50 mM Tris PH7.6150 mM NaCl, 1% vol/vol NP‐40, and complete protease inhibitors), followed by centrifugation at 17000 g for 10 min at 4°C. Cell lysates were purified with anti‐Flag affinity beads and eluted with FLAG peptides. The eluates were resolved on silver staining. The results were subjected to mass spectrometry.

### 2.11 GST pull down assay

The plasmid for GST‐PDLIM3 was transfected into *E*. *coli*. The fusion proteins were prepared as described previously.^27^ Approximately 100 μg of GST and GST‐PDLIM3 fusion protein was immobilized in 50 μL of glutathione agarose and equilibrated before being incubated together at 4°C for 60 min with gentle rocking motion. Subsequently, anti‐NAA40 was incubated with the immobilized GST‐PDLIM3 and GST at 4°C overnight under gentle rotation. The bound proteins were eluted with elution buffer (10 mM glutathione in PBS, pH 8.0) and analyzed by western blot.

### 2.12 Ubiquitination assay

To detect ubiquitinated‐NAA40, THP‐1‐DM cells were transiently transfected with HA‐NAA40 or Flag‐PDLIM3 as indicated. For immunoprecipitation (IP), whole cell lysates were prepared as above. Cell lysates (2000 μg) were incubated for overnight at 4°C with 50 μg of either mouse anti‐HA antibody or mouse IgG (Sigma‐Aldrich) cross‐linked to protein A/G beads (Pierce Crosslink IP Kit, Life Technologies). The immune complexes were collected by centrifugation, washed five times in IP lysis buffer, and eluted with glycine. The eluant were resolved by SDS/PAGE and probed by anti‐Ubiquitin antibody (#ab7780, Abcam).

### 2.13 Enzyme‐linked immunosorbent assay (ELISA)

The secretion of human IL10, TGF‐β, and CCL22 by THP‐1‐DM or hMDM were detected via respective ELISA kit (Boster) following the manufacturer's instructions, and the absorbance at 450 nm was measured.

### 2.14 Chromatin immunoprecipitation (ChIP)

For ChIP assay, cells were subjected to 1% formaldehyde at room temperature for 10 min, followed by the addition of 0.125 M glycine to terminate the crosslinking of chromatin proteins to DNA. Then, cells were lysed and the resulting lysate was sonicated to obtain 300∼500 bp chromatin fragments. Following immunoprecipitation with anti‐NAA40, anti‐N‐acH4, anti‐H3K4me3, anti‐H3K27me3, anti‐DNMT1, anti‐DNMT2, anti‐DNMT3a, anti‐DNMT3b, anti‐DNMT3L, or anti‐TET, the immunoprecipitated DNA fragments were purified and tested via RT‐qPCR. The groups immunoprecipitated with anti‐IgG served as negative controls.

### 2.15 Methylation‐specific PCR (MSP)

The methylation levels of PDLIM3 promoter were estimated by MSP. First, genomic DNA was isolated and Methylation‐specific primers were obtained. Then, 10 μL of the MSP reaction was mixed by 4 μL modified DNA template, 0.6 μL methylation‐specific or nonmethylation‐specific primers, 1 μL 10X Buffer I, 0.1 μL HsTaq DNA polymerase mixture, and 3.5 μL ddH_2_O. The PCR were performed with 95°C for 5 min followed by 35 cycles of 95°C for 30 s, 63°C for 30 s, and 72°C for 30 seconds, and then 72°C for 10 min. Finally, the PCR samples were separated and analyzed by 2% agarose gel electrophoresis (AGE), with the gel containing ethidium bromide for sample staining.

### 2.16 In vivo study

For animal study, 30 eight‐week‐old female Sprague–Dawley (SD) rats weighing 240–260 g were purchased from Shanghai Lingchang Biotechnology. After being approved by the Medical Ethics Committee of the First Affiliated Hospital of Soochow University, experimental procedures of animal study were conducted in accordance with animal care and use guideline of the Institutional Animal Experimental Ethnics Committee. The rats were anesthetized with 4% isoflurane, then made a small incision at the center of abdomen. Excised the left uterine horn and placed it into saline solution. Dissected the endometrium from muscles, then cut into pieces (5 mm in each dimension). Made a subcutaneous pocket on the abdominal wall, then placed the uterine fragment in the pocket facing the abdominal muscle. Rats were then subcutaneously injected with 30 μg/kg benzoic estradiol immediately after surgery, and the treatment lasted for 10 days. After successfully establishing the rat Ems model, these rats were randomly divided into six groups (Sham, Ems, Ems + ecESCs‐Exos, Ems + ecESCs/sh‐1‐Exos, Ems + ecESCs‐Exos + control liposomes, Ems + ecESCs‐Exos + clodronate liposomes; *n* = 5 per group). The sham group underwent surgery without autotransplantation of endometrial tissues. Exosomes (50 μg per rat) were administered via intraperitoneal injection every five days for a total of five injections. The exosome dose was normalized to total protein content. For macrophages clearance, rats were treated with clodronate liposomes (20 mg/kg) through tail vein injection, once a week, for 4 weeks. Above interventions (including exosomes or liposomes injections) were started at 17 days after benzoic estradiol treatment. Rats were randomized to treatment groups, and lesion measurement and histological analysis were performed by investigators blinded to group allocation. The body weight and ectopic endometrial cyst volume were monitored every 3 days. The volume of ectopic endometrial cyst was calculated using the formula *V* = 1/2 (L × W^2^). The paraffin‐embedded tissue samples were cut into 20 μm thick sections for hematoxylin‐eosin staining following the standard protocol.

### 2.17 Statistical analysis

All the experiments were repeated a minimum of three times. The statistical analyses were performed using SPSS software with Student's *t* test or one‐way ANOVA. Data were plotted as the mean ± SD for visualization. Data with *p* value < 0.05 was defined as significant data.

## 3 RESULTS

### 3.1 M2 macrophages promote the proliferative and migratory phenotypes of ecESCs

We induced the differentiation of THP‐1 monocyte line into macrophages (namely THP‐1‐DM) by phorbol 12‐myristate 13‐acetate (PMA), and then treated with IL‐4 to induce the M2 phenotype of macrophages. The expression of M2 phenotypic marker CD206 was detected by flow cytometry. The results showed that the expression of CD206 in THP‐1‐DM cells was significantly increased by IL‐4 treatment (Figure 1A), indicating successful induction of M2 macrophages in this way. After co‐culture of M2‐polarized macrophages with ecESCs cells, CCK8 was used to detect ecESCs cell vitality before and after co‐culture, EdU was used to detect cell proliferation, and Transwell migration assay was used to analyze cell transfer ability. The results showed that the activity, proliferation and migration ability of ecESCs cells were significantly increased after co‐culture (Figure 1B–D). Moreover, we found that M2 macrophage markers in ectopic endometrial tissues were significantly higher than those in normal endometrial tissues by immunofluorescence assay (Figure 1E). When ecESCs cells were co‐cultured with THP‐1‐DM cells, the expressions of M2 polarization markers (including CD163, ARG1 and MRC1) in macrophages were analyzed by RT‐qPCR and western blot, and the results showed that the expressions of CD163, ARG1 and MRC1 in THP‐1‐DM cells were significantly increased after co‐culture (Figure 1F–G). Also, the levels of several cytokines secreted by M2 macrophages were markedly elevated in conditioned medium of THP‐1‐DM cells after co‐culture (Figure 1H).

**FIGURE 1:**
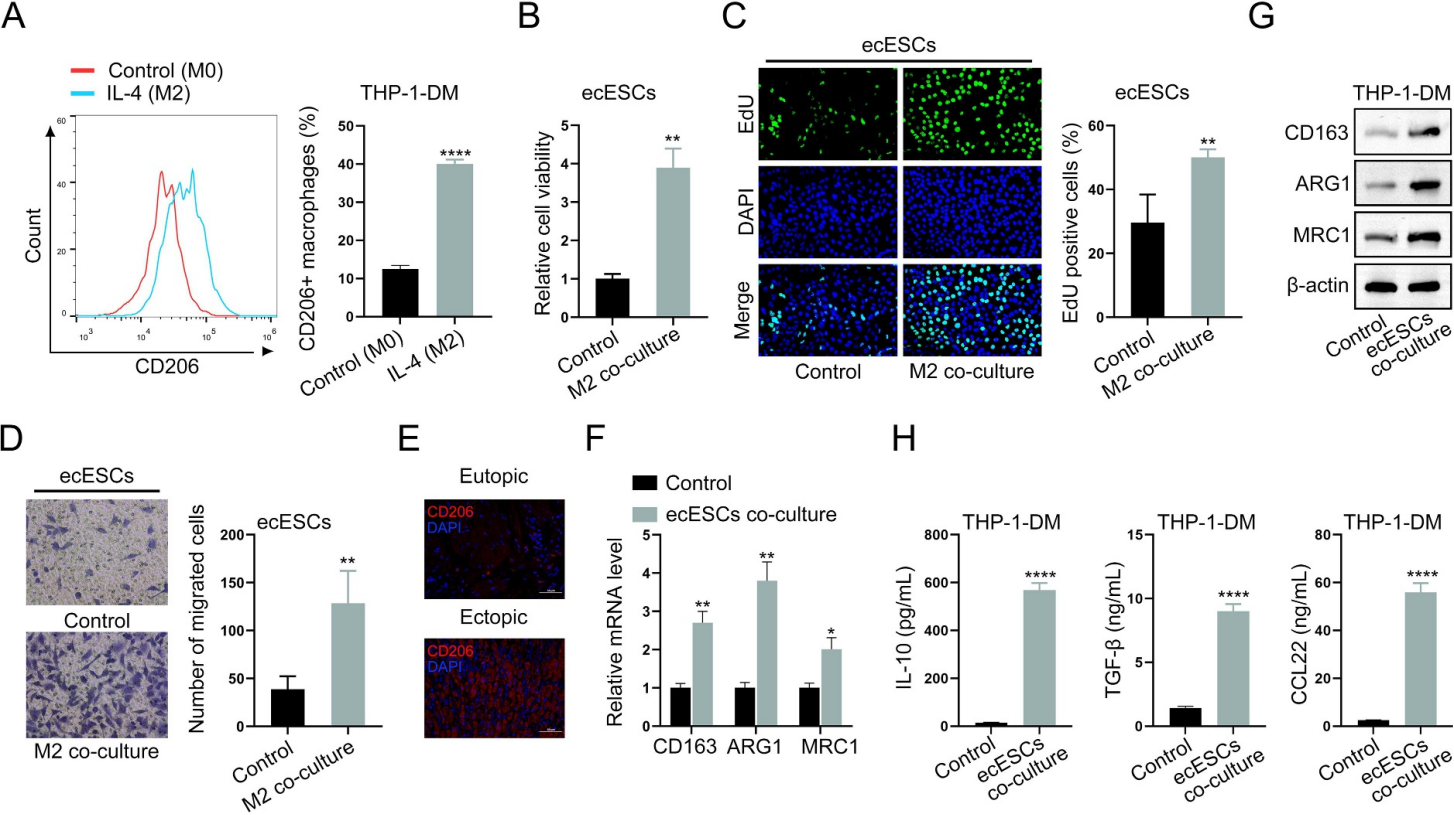
M2 macrophages promote the proliferative and migratory phenotypes of ecESCs. (A) The expression of CD206 in THP‐1‐DM cells induced by IL‐4 was detected by flow cytometry. (B) The activity of ecESCs cells before and after co‐culture of M2 macrophages was analyzed by CCK‐8. (C) EdU analyzed the proliferation ability of ecESCs cells before and after co‐culture of M2 macrophages. (D) The migration ability of ecESCs cells before and after M2 macrophage co‐culture was analyzed by transwell. (E) The content of CD206 in orthotopic endometrial tissue and ectopic endometrial tissue was analyzed by immunofluorescence. (F) Constructed ecESCs cells were co‐cultured with THP‐1‐DM cells, and the expressions of CD163, ARG1, and MRC1 in THP‐1‐DM cells were detected by RT‐qPCR before and after co‐culture. (G) The expressions of CD163, ARG1, and MRC1 in THP‐1‐DM cells before and after co‐culture were detected by western blot. (H) The levels of IL‐10, TGF‐β, and CCL22 secreted by THP‐1‐DM cells before and after co‐culture were examined via ELISA. *p < 0.05, **p < 0.01, ****p < 0.0001.

### 3.2 PDLIM3 promotes macrophage M2 polarization and ecESCs development

To further analyze the specific mechanisms of Ems development, we obtained four datasets from the GEO database (https://www.ncbi.nlm.nih.gov/gds/). Through intersecting the four datasets, we found that PDLIM3 is upregulated in ectopic endometrial tissues compared with the eutopic endometrial tissues (Figure 2A). The expression of PDLIM3 in nESCs and ecESCs cells was detected by RT‐qPCR and western blot. The results showed that the expression of PDLIM3 in ecESCs cells was significantly increased (Figure 2B). Recently, PDLIM3 is reported to be a potential biomarker for immune infiltration in Ems patients and is associated with macrophage M2 polarization.^22^ In order to determine whether Ems promotes the M2 polarization of macrophages through PDLIM3, we first analyzed the correlation between the expression of PDLIM3 and M2 macrophages, and the results showed that high expression of PDLIM3 was positively correlated with M2 polarization (Figure 2C). After knocking down PDLIM3 in ecESCs cells (Figure S1A), we co‐cultured THP‐1‐DM cells with ecESCs cells under PDLIM3 interference or not, and detected the changes in expressions of CD163, ARG1 and MRC1 in THP‐1‐DM cells. The data of RT‐qPCR and western blot showed that compared to control group, the expressions of CD163, ARG1, and MRC1 in THP‐1‐DM cells were significantly enhanced after co‐culture with ecESCs, whereas it recovered after co‐culture with PDLIM3‐silenced ecESCs (Figure 2D,E). Similar changes were also observed in the levels of IL‐10, *β* and CCL22 secreted by THP‐1‐DM cells (Figure 2F). Flow cytometry also showed that the expression of CD206 in THP‐1‐DM cells increased after co‐culture, but decreased after co‐culture with ecESCs cells interfering with PDLIM3 (Figure 2G). These results indicate that PDLIM3 is a key gene in promoting M2 polarization in macrophages.

**FIGURE 2:**
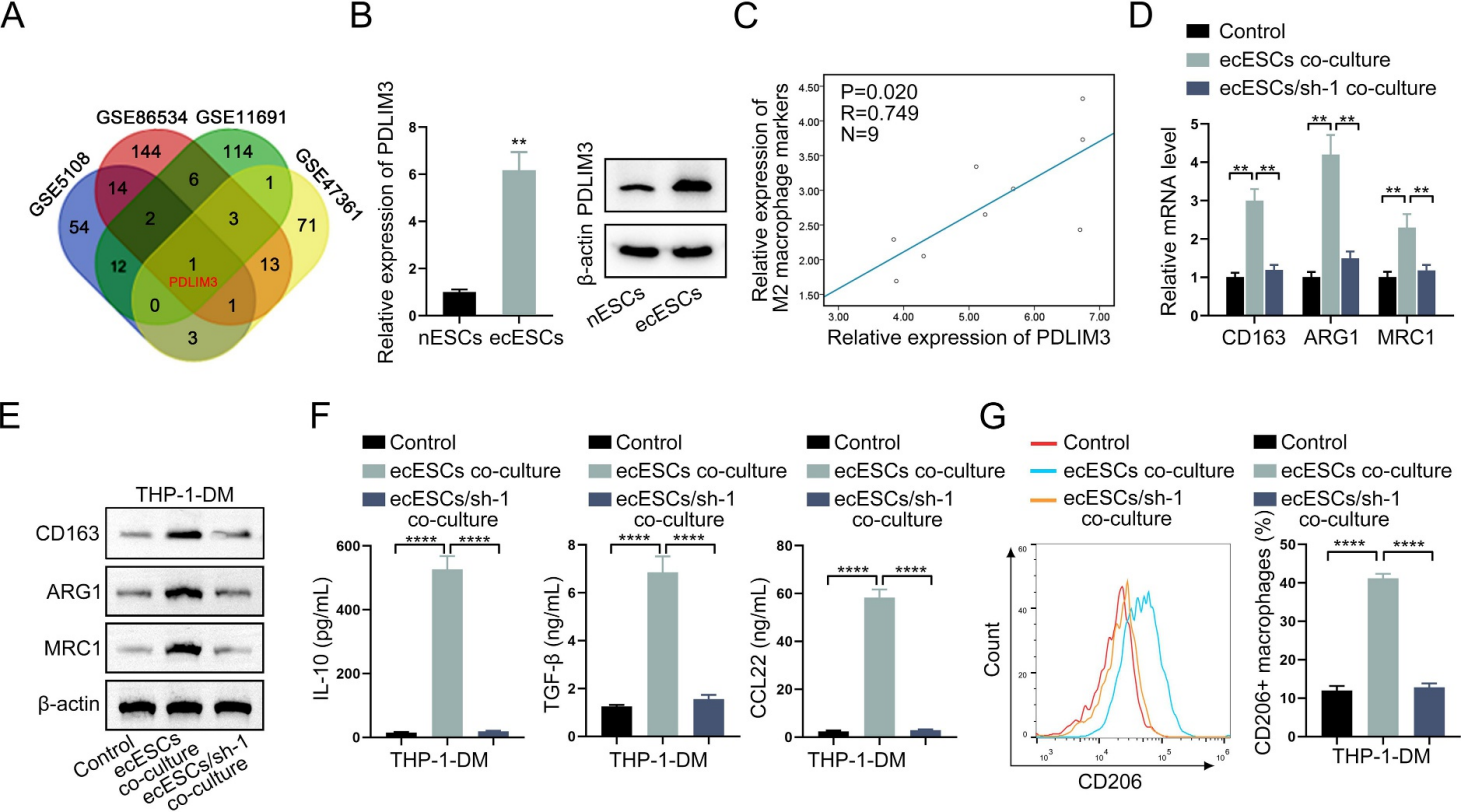
PDLIM3 promotes macrophage M2 polarization. (A) The mRNA associated with EC progression was screened out through intersecting the four datasets obtained from GEO database. (B) Both mRNA and protein levels of PDLIM3 were measured in ecESCs and normal endometrial stromal cells by RT‐qPCR and western blot. (C) Correlation analysis of PDLIM3 expression and the expression of M2 markers in macrophages. (D) The effect of interfering PDLIM3 in ecESCs on CD163, ARG1, and MRC1 mRNA expression in THP‐1‐DM cells after co‐culture was analyzed by RT‐qPCR. (E) The effect of interfering PDLIM3 in ecESCs on CD163, ARG1, and MRC1 protein expression in THP‐1‐DM cells after co‐culture was analyzed by western blot. (F) The effect of interfering PDLIM3 in ecESCs on the secretion of IL‐10, TGF‐β, and CCL22 by THP‐1‐DM after co‐culture was analyzed using ELISA. (G) The effect of interfering PDLIM3 in ecESCs on CD206 expression in THP‐1‐DM cells after co‐culture was analyzed by flow cytometry. **p < 0.01, ****p < 0.0001.

Moreover, data of CCK‐8 assay revealed that the viability of ecESCs was reduced after silencing of PDLIM3 (Figure S1B). The results of EdU assay demonstrated that knockdown of PDLIM3 in ecESCs suppressed cell proliferation (Figure S1C). The migrating ability of ecESCs was also inhibited in face of PDLIM3 knockdown (Figure S1D). The same experiments were conducted in nESCs to make further confirmation. Considering the low expression level of PDLIM3 in nESCs, we overexpressed it for gain‐of‐function assays (Figure S1E). According to experimental results, overexpression of PDLIM3 in nESCs enhanced cell viability (Figure S1F), promoted cell proliferation (Figure S1G) and accelerated cell migration (Figure S1H). These results show that PDLIM3 facilitates proliferation and migration of ecESCs.

### 3.3 ecESCs‐secreted exosomes transfer PDLIM3 to induce macrophage M2 polarization

Studies have shown that exosomes are important cell function regulators in Ems.^28^ Moreover, a previous study revealed that exosomes derived from Ems mouse model induced macrophage M2 polarization.^21^ Our previous studies demonstrated that PDLIM3 induced M2 polarization of macrophages in ecESCs. Given the important role of exosomes in M2 polarization of endogenic microenvironment, we attempted to explore whether PDLIM3 entered THP‐1‐DM through the exosomal pathway. At first, we co‐cultured ecESCs and THP‐1‐DM and measured the expression changes of M2 markers. As indicated in RT‐qPCR and western blot data, the expressions of M2 markers were upregulated at both mRNA and protein levels in THP1‐DM co‐cultured with ecESCs, whereas these tendencies were abolished by adding the exosome secretion inhibitor GW4869 (Figure 3A,B). Besides, the enhanced secretions of IL‐10, TGF‐β and CCL22 by THP‐1‐DM co‐cultured with ecESCs were reversed after GW4869 treatment (Figure 3C). In the meantime, we detected CD206‐positive macrophages to analyze the percentage of M2 macrophages. In consistent with the above data, co‐culturing with ecESCs increased the percentage of M2 macrophages, whereas GW4869 treatment abolished the increased tendency (Figure 3D). Therefore, we supposed that ecESCs might induce macrophage M2 polarization by secreting exosomes. To demonstrate our supposition, we isolated exosomes from ecESCs and observed them under a TEM (Figure 3E). The surface markers of exosomes (CD81 and TSG101) were detected in ecESCs‐Exos, whereas the negative marker calnexin was undetected (Figure 3F). The diameter and concentration of exosomes were analyzed by NTA (Figure 3G). The exosomes were also labeled by PKH26 staining for tracing (Figure 3H). To explore whether PDLIM3 was delivered by ecESCs‐Exos to macrophages, we treated THP1‐DM with ecESCs‐Exos and detected the significant upregulation of PDLIM3 mRNA and protein (Figure 3I). Importantly, the increased protein level of PDLIM3 in THP1‐DM cells induced by ecESCs‐Exos was reversed when silencing PDLIM3 in ecESCs (Figure 3J), indicating that the upregulation of PDLIM3 in THP‐1‐DM was attributed by direct transfer of PDLIM3 via ecESCs‐Exos. Moreover, we discovered that although ecESCs‐Exos contained both PDLIM3 mRNA and protein (Figure S2A,B), the ratio of PDLIM3 protein/mRNA was relatively unchanged in THP‐1‐DM after ecESCs‐Exos treatment (Figure S2C). Besides, it showed that both the augmented PDLIM3 mRNA and protein levels in THP‐1‐DM induced by ecESCs‐Exos were not impacted in response to protein synthesis inhibitor CHX (Figure S2D,E). These data evidenced that ecESCs‐Exos directly transferred PDLIM3 protein to THP‐1‐DM cells. Subsequently, we measured M2 markers and M2 secreted cytokines in indicated macrophages. It was uncovered that both the expressions of M2 markers and the levels of M2 secreted cytokines were enhanced in THP1‐DM treated with ecESCs‐Exos, whereas such phenomena were countervailed when treated with PDLIM3‐silenced ecESCs‐derived exosomes (Figure 3K–M). Consistently, the percentage of CD206‐positive macrophages was increased in THP1‐DM treated with ecESCs‐Exos but remain unchanged when treated with exosomes derived from PDLIM3‐silenced ecESCs (Figure 3N). Furthermore, we validated the findings in primary hMDM. Similarly, data showed that ecESCs‐Exos treatment contributed to M2 polarization of hMDM via transferring PDLIM3, since the levels of M2 markers and M2 secreted cytokines as well as the percent of CD206‐positive macrophages increased by ecESCs‐Exos were all reversed when silencing PDLIM3 in ecESCs (Figure S2F–H). According to these data, we confirm that exosomal PDLIM3 derived from ecESCs to induce macrophage M2 polarization.

**FIGURE 3:**
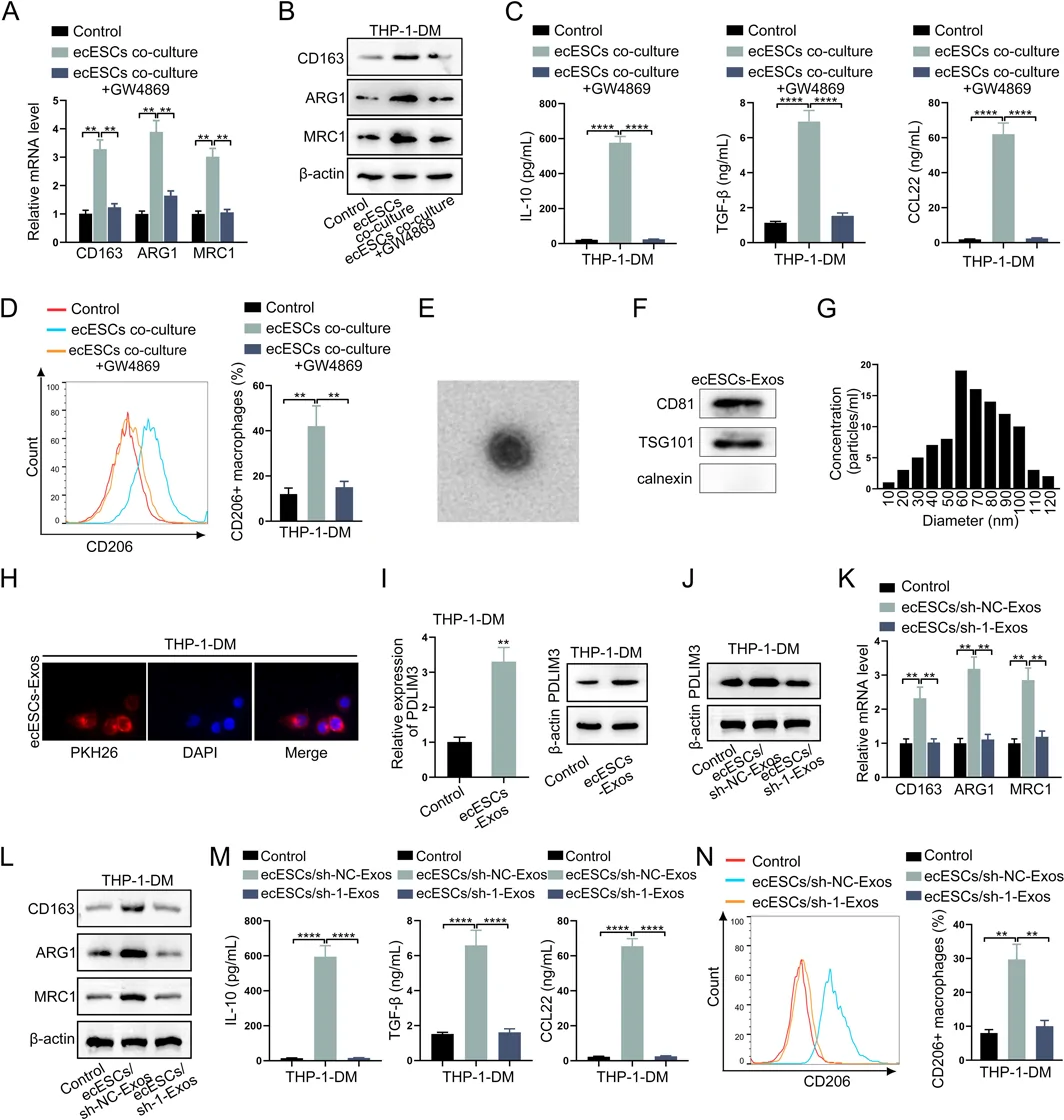
ecESCs‐secreted exosomes transfer PDLIM3 to induce macrophage M2 polarization. (A, B) The mRNA and protein levels of M2 markers were measured in THP‐1‐DM co‐cultured with ecESCs or ecESCs and GW4869 (the exosome secretion inhibitor) by RT‐qPCR and western blot. (C) The levels of IL‐10, TGF‐β, and CCL22 were evaluated by ELISA in THP‐1‐DM with or without ecESCs co‐culture or further GW4869 supplement. (D) The percentage of CD206‐positive macrophages was measured by flow cytometry in different co‐culture systems. (E) Exosomes isolated from ecESCs were observed under a TEM. (F) The surface markers of exosomes (CD81 and TSG101) and the negative marker (calnexin) were detected in ecESCs‐Exos by western blot. (G) The diameter and concentration of exosomes were analyzed by nanoparticle tracking analysis. (H) The exosomes were also labeled by PKH26 staining for tracing. (I) The level of PDLIM3 mRNA and protein was detected in THP‐1‐DM treated with ecESCs‐Exos by RT‐qPCR and western blot. (J) The protein level of PDLIM3 was measured in THP‐1‐DM treated with exosomes secreted by control or PDLIM3‐silenced ecESCs by western blot. (K, L) The mRNA and protein levels of M2 markers were measured in THP‐1‐DM treated with exosomes from control or PDLIM3‐silenced ecESCs by RT‐qPCR and western blot. (M) ELISA analyzed the secretion of IL‐10, TGF‐β, and CCL22 by THP‐1‐DM treated with exosomes from control or PDLIM3‐silenced ecESCs. (N) The percentage of CD206‐positive macrophages was measured in THP‐1‐DM treated with ecESCs‐Exos or PDLIM3‐silenced ecESCs‐derived exosomes by flow cytometry. **p < 0.01, ****p < 0.0001.

### 3.4 PDLIM3 interacts with NAA40 to stabilize NAA40 protein

Next, we investigated the regulatory mechanism of PDLIM3 in macrophages. We performed Co‐IP assay and analyzed the results through mass spectrometry. As shown in Figure 4A, NAA40 is a PDLIM3‐interacting protein. The interaction between PDLIM3 and NAA40 was also validated in THP1‐DM by Co‐IP assay and detected by western blot (Figure 4B). We then explored whether PDLIM3 affected NAA40 protein expression. The results in Figure 4C illustrated that overexpression of PDLIM3 could enhance NAA40 protein level. To analyze the specific interacting mechanism between PDLIM3 and NAA40, we analyzed the structure of PDLIM3 and divided it into different parts (Figure 4D). Through GST‐pull down assay, we determined that NAA40 interacted with PDLIM3 at part 184–293 (Figure 4E). Next, we detected NAA40 protein levels in THP‐1‐DM treated with CHX, MG132, and BafA1 at different time intervals after overexpression of PDLIM3. It showed that overexpression of PDLIM3 extended the half‐life of NAA40 protein in THP‐1‐DM under CHX treatment (Figure 4F), suggesting PDLIM3 retarded NAA40 degradation. Interestingly, such extension could be reversed by MG132 but not by BafA1 (Figure 4G), evidencing that PDLIM3 inhibited the degradation of NAA40 protein in proteasome‐mediated way. Herein, we measured the ubiquitination level of NAA40 protein with the increasing of Flag‐PDLIM3 and the result indicated that PDLIM3 suppressed the ubiquitination of NAA40 protein in a dose‐dependent manner (Figure 4H). Resultantly, the final level of HA‐NAA40 in THP‐1‐DM increased with the dose of Flag‐PDLIM3 (Figure 4I). In summary, PDLIM3 directly interacts with NAA40 and enhances NAA40 protein stability by decreasing its ubiquitination.

**FIGURE 4:**
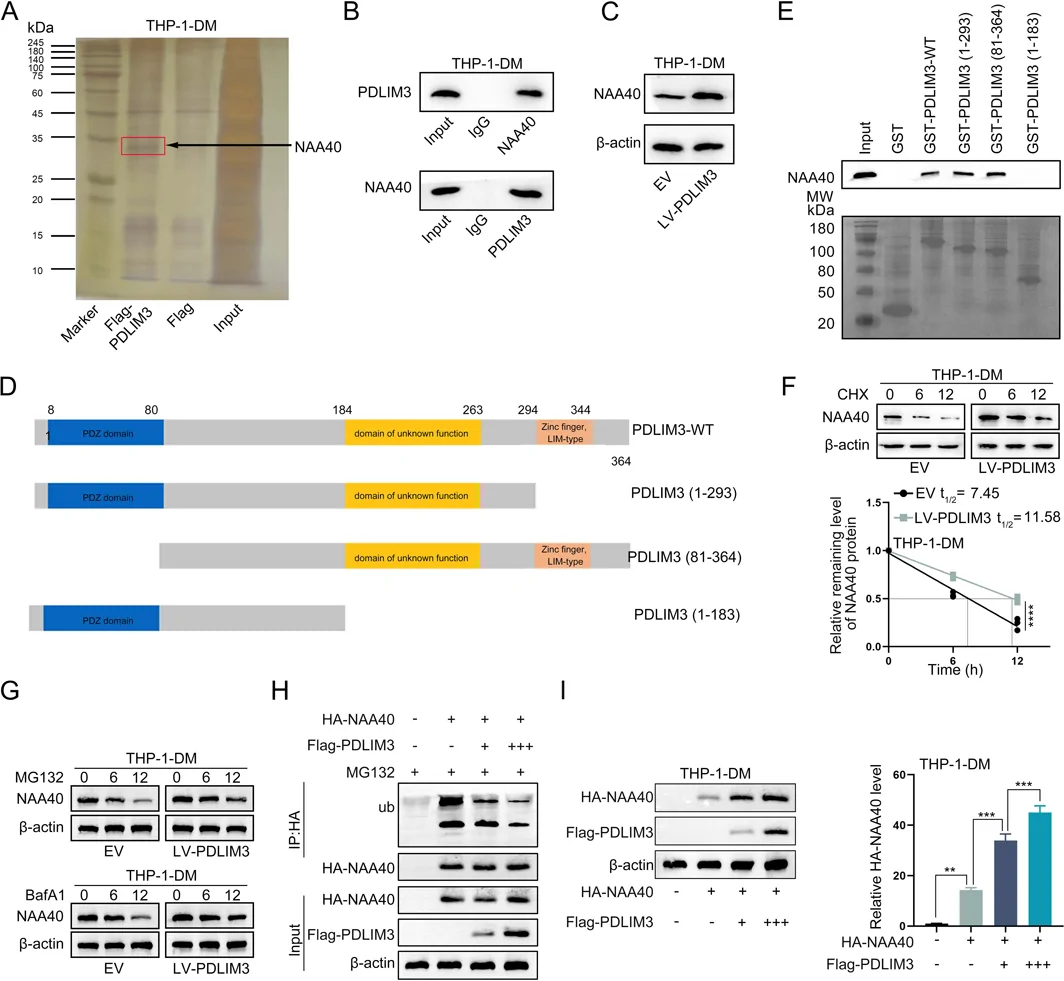
PDLIM3 interacts with NAA40 to stabilize NAA40 protein. (A) Proteins interacting with PDLIM3 were detected by IP assay and analyzed via mass spectrometry. (B) The interaction between PDLIM3 and NAA40 was also validated in macrophages by Co‐IP assay and detected by western blot. (C) Overexpression of PDLIM3 could enhance NAA40 protein level, as detected by western blot. (D) The structure of PDLIM3 was analyzed and divided into different parts. (E) NAA40 interacted with PDLIM3 at part 1834‐293 was determined by GST‐pull down assay. (F) The impact of PDLIM3 overexpression on the protein levels of NAA40 were analyzed by western blot in THP‐1‐DM under treatment with CHX for 0, 6, 12 h, with the degradation curves were obtained after quantification. (G) The protein levels of NAA40 in control or PDLIM3‐overexpressed THP‐1‐DM cells treated with MG132 and bafilomycin A1 for different time intervals were detected by western blot. (H) The ubiquitination level of HA‐NAA40 protein in MG132‐treated THP‐1‐DM cells with the increasing of Flag‐PDLIM3 we measured by ubiquitination assay. (I) The changes in HA‐NAA40 protein level in THP‐1‐DM cells with different dose of Flag‐PDLIM3 were analyzed via western blot and then quantified. **p < 0.01, ***p < 0.001, ****p < 0.0001.

### 3.5 NAA40 activates PRMT1 to promote macrophage M2 polarization

According to reports, NAA40‐mediated N‐terminal acetylation of histone H4 (N‐acH4) controls gene expression by altering the deposition of histone H4 arginine 3 asymmetric dimethylation (H4R3me2a).^29^, ^30^ PRMT1 is a member of protein–arginine methyltransferases, which is the most abundant one in mammals. PRMT1 is mainly responsible for catalyzing H4R3me2a to transcriptionally activating target genes. Here, we detected whether NAA40 regulate PRMT1 to affect macrophage M2 polarization. Before mechanism exploration, we knocked down NAA40 expression using specific shRNAs (Figure 5A). Next, we found that the levels of N‐acH4 and H4R3me2a were significantly decreased after silencing of NAA40 (Figure 5B). We then uncovered that NAA40 knockdown led to the downregulation of both PRMT1 mRNA and protein (Figure 5C,D). Moreover, ChIP‐qPCR data proved the abundant occupancy of NAA40 in PRMT1 promoter (Figure 5E). Meanwhile, the N‐acH4 level in PRMT1 promoter was reduced a lot after NAA40 expression was knocked down (Figure 5F). Furthermore, we detected the decreased H3K4me3 level and the increased H3K27me3 level in PRMT1 promoter after silencing of NAA40 (Figure 5G,H). To detect the role of NAA40‐mediated PRMT1 activation in macrophage M2 polarization, we overexpressed PRMT1 (Figure 5I) and conducted rescue assays in THP1‐DM treated with ecESCs‐Exos. As indicated in Figure 5J,K, the expression levels of M2 markers were all reduced after silencing of NAA40 but was restored after co‐transfection with PRMT1 overexpression vector. The same tendencies were observed in the levels of secreted cytokines and the percent of CD206‐positive macrophages (Figure 5L,M). Collectively, NAA40‐mediated H4R3me2a activates PRMT1 to promote macrophage M2 polarization.

**FIGURE 5:**
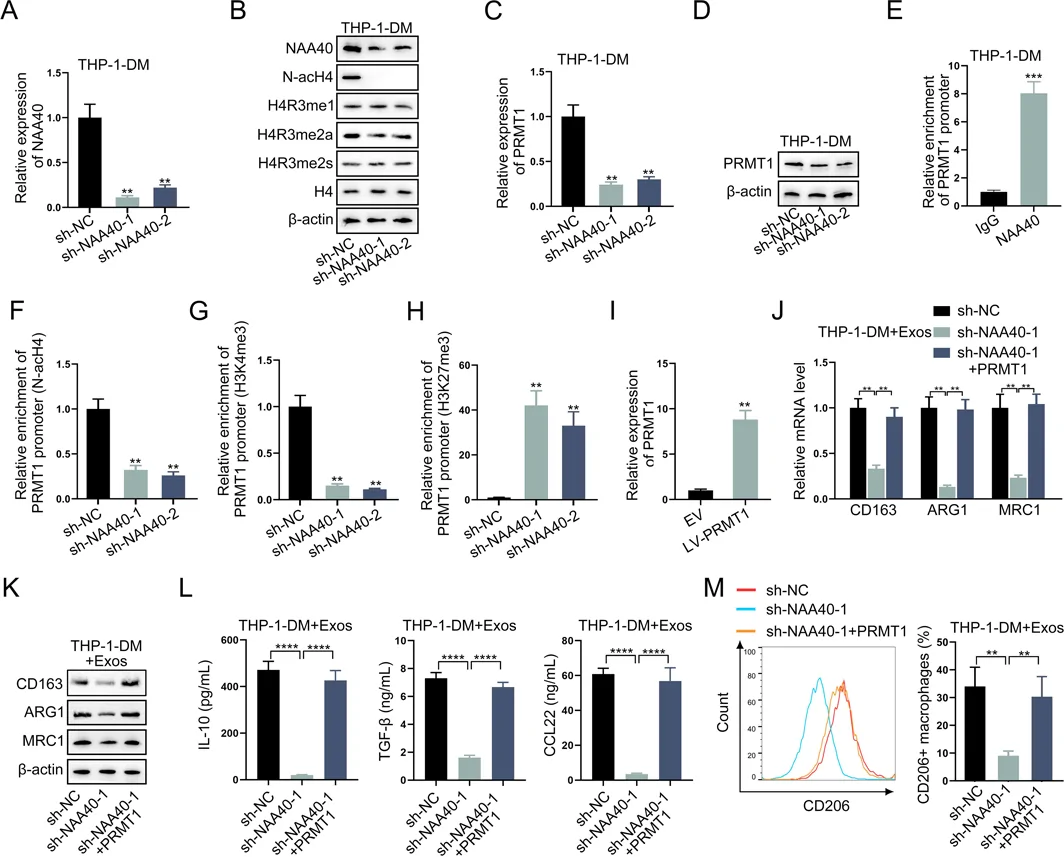
NAA40 activates PRMT1 to promote macrophage M2 polarization. (A) NAA40 expression was knocked down using specific shRNAs. (B) The levels of N‐acH4, H4R3me1, H4R3me2a, and H4R3me2s were detected in THP‐1‐DM after silencing of NAA40. (C, D) The expression of PRMT1 mRNA and protein was measured by RT‐qPCR and western blot in THP‐1‐DM with NAA40 knockdown or not. (E) ChIP‐qPCR detected the binding of NAA40 to PRMT1 promoter in THP‐1‐DM cells. (F) The N‐acH4 level in PRMT1 promoter was detected by ChIP‐qPCR in THP‐1‐DM before or after NAA40 expression was knocked down. (G, H). The decreased H3K4me3 level and the increased H3K27me3 level were detected in PRMT1 promoter by ChIP‐qPCR in THP‐1‐DMwith or without silencing of NAA40. (I) PRMT1 was overexpressed in THP‐1‐DM and the overexpression efficiency was determined by RT‐qPCR. (J, K) The expression levels of M2 markers were all reduced after silencing of NAA40 but was restored after co‐transfection with PRMT1 overexpression vector, as detected by RT‐qPCR and western blot. (L) ELISA detected the levels of several cytokines secreted by indicated THP‐1‐DM cells. (M) The percentage of CD206‐positive macrophages was reduced after silencing of NAA40 but was restored after co‐transfection with PRMT1 overexpression vector, as detected by flow cytometry. **p < 0.01, ***p < 0.001, ****p < 0.0001.

### 3.6 Exosomal PDLIM3 promotes ESCs growth and migration through upregulating PRMT1 to induce macrophage M2 polarization

Rescue assays were carried out to confirm the effect of exosomal PDLIM3‐induced M2 polarization of macrophages on the functions of ESCs. As detected by CCK‐8 and EdU assays, the viability and proliferation of ecESCs promoted by ecESCs‐derived exosomes‐treated THP1‐DM was suppressed by PDLIM3 silencing but was recovered again by overexpression of PRMT1 (Figure S3A,B). Similarly, the migration of ecESCs promoted by ecESCs‐derived exosomes‐treated THP1‐DM was inhibited after knockdown of PDLIM3 but was restored after overexpression of PRMT1 (Figure S3C). The same experiments were carried out in nESCs and the results indicated that viability, proliferation and migration of nESCs promoted by ecESCs‐derived exosomes‐treated THP1‐DM was inhibited after knockdown of PDLIM3 but was restored after overexpression of PRMT1 (Figure S3D–F). Therefore, we conclude that exosomal PDLIM3‐induced macrophage M2 polarization promotes ESCs’ growth and migration by activating PRMT1.

### 3.7 PDLIM3 mediated by the DNA methyltransferase DNMT1 is upregulated in ecESCs

In addition, we also studied the possible mechanism of PDLIM3 upregulation in ectopic endometrium. According to GSE87621 dataset, DNA methylation of PDLIM3 was decreased in ESCs isolated from ectopic endometrial tissues compared with those from normal endometrial tissues (Figure 6A, the upper panel). Meanwhile, through searching on MethPrimer website (http://www.urogene.org/methprimer/index.html), there are two CpG islands in PDLIM3 promoter (Figure 6A, the lower panel). These data indicated that upregulation of PDLIM3 in ecESCs might be induced by the decreased DNA methylation level in its promoter. To identify the mechanism inducing PDLIM3 upregulation in ecESCs, we performed MSP and AGE detection and found that the DNA methylation level of PDLIM3 promoter in ecESCs was significantly lower than that in nESCs (Figure 6B). In order to determine whether passive demethylation or active demethylation is necessary, we detected the expression of methylase and demethyltransferase in nESCs and ecESCs, and the results showed that the expression of DNMT1 was significantly reduced in ecESCs compared to nESCs (Figure 6C). ChIP‐qPCR results also showed that DNMT1 was enriched in the PDLIM3 promoter region (Figure 6D). To determine the regulatory effect of DNMT1 on PDLIM3 expression, we overexpressed DNMT1 in ecESCs (Figure 6E). Subsequently, we detected PDLIM3 mRNA expression and found the decreased mRNA level under DNMT1 overexpression (Figure 6F). Finally, we detected the decreased DNA methylation level of PDLIM3 promoter in DNMT1‐overexpressed ecESCs through MSP and AGE assays (Figure 6G). These results suggest that PDLIM3 is upregulated in ecESCs due to the DNMT1 downregulation‐mediated reactivation of PDLIM3.

**FIGURE 6:**
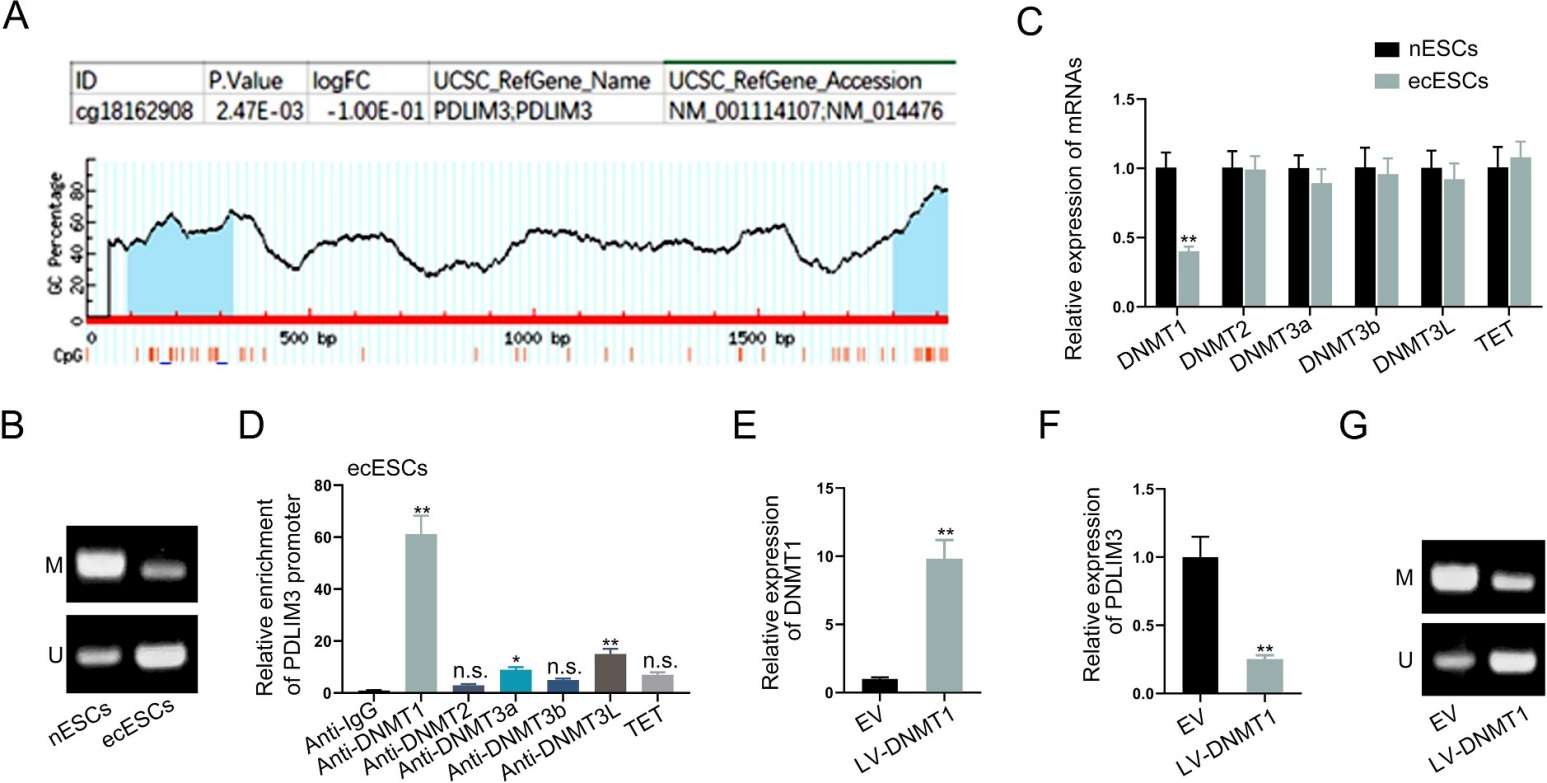
PDLIM3 upregulation is mediated by the DNA methyltransferase DNMT1 in ecESCs. (A) GSE87621 dataset revealed the DNA methylation level of PDLIM3 promoter in endometrial stromal cells isolated from ectopic endometrial tissues or normal endometrial tissues (upper). Two CpG islands in PDLIM3 promoter were found by searching on MethPrimer website (lower). (B) MSP detection revealed the difference in the methylation levels of PDLIM3 promoter in ecESCs and nESCs. (C) The expression of DNA methyltransferase and demethyltransferase in ecESCs and nESCs was analyzed by RT‐qPCR. (D) The binding of DNA methylation regulators to PDLIM3 promoter in ecESCs was detected by ChIP assay. (E) RT‐qPCR validated that DNMT1 was overexpressed in ecESCs after transfection with DNMT1 overexpression vector. (F) PDLIM3 mRNA expression was detected by RT‐qPCR in ecESCs with or without overexpression of DNMT1. (G) The DNA methylation level of PDLIM3 promoter was measured in control or DNMT1‐overexpressed ecESCs through MSP. *p < 0.05, **p < 0.01; n.s. = not significant. MSP, methylation‐specific PCR; nESCs, normal endometrial stromal cells.

### 3.8 Exosomal PDLIM3 promotes ectopic endometrium formation and macrophage M2 polarization in rat model

Animal model was established to demonstrate the role of exosomal PDLIM3 in Ems and whether its function relied on macrophage M2 polarization. As shown in Figure 7A, no significant difference of body weight between different groups. As shown in Figure 7B, the ectopic endometrium cyst volume of Ems rats was enhanced compared with Sham mice, and it was further increased after injection of ecESCs‐Exos; but it was reversed under PDLIM3 silence or macrophage elimination via clodronate liposomes. Meanwhile, Hematoxylin‐eosin staining revealed that the ectopic endometrium formation increased in Ems rats was further enhanced by injection of ecESCs‐Exos, whereas such enhancement was counteracted when silencing PDLIM3 in ecESCs or eliminating macrophages of rats (Figure 7C). We also analyzed the macrophage M2 polarization in different rat models. Through assessing changes in the expression levels of M2 markers and the percentage of CD206‐positive macrophages, we confirmed that ecESCs‐Exos could induce macrophage M2 polarization, but silencing of PDLIM3 abolished the effect of ecESCs‐Exos, not to mention after removing macrophage via clodronate liposomes (Figure 7D–F). The above results indicate that ecESCs‐derived exosomal PDLIM3 promotes ectopic endometrium formation via inducing macrophage M2 polarization in rat model.

**FIGURE 7:**
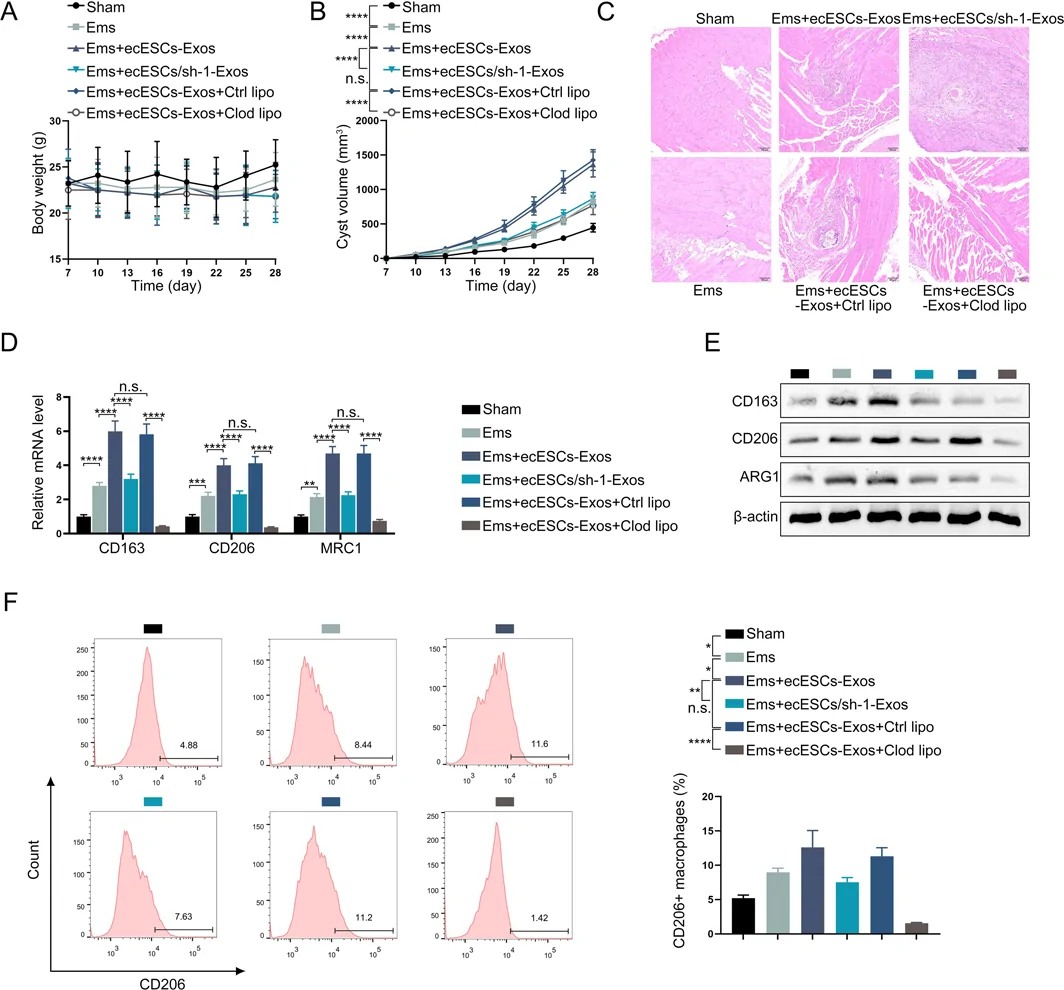
Exosomal PDLIM3 promotes ectopic endometrium formation and macrophage M2 polarization in rat model. (A) No significant difference of body weight between different groups. (B) The ectopic endometrium cyst volume of different groups of rat models was measured. (C) Hematoxylin‐eosin staining revealed the ectopic endometrium formation in different groups of rat models. (D, E) The expression levels of M2 markers were measured in different groups of rat models by RT‐qPCR and western blot. (F) The percentage of CD206‐positive macrophages was measured in different groups of rat models by flow cytometry. *p < 0.05, **p < 0.01, ****p < 0.0001; n.s. = not significant.

## 4 DISCUSSION

DNA methylation is a key epigenetic reprogramming mechanism that modulate RNA expression.^31^, ^32^ According to the GSE5108, GSE86534, GSE11691, and GSE47361 data sets, we obtained significantly high expression of PDLIM3 in Ems tissues, and the results of GSE87621 data set showed that PDLIM3 DNA methylation was reduced in ESCs. Methyltransferases (DNMTs) are main regulators in processing DNA methylation.^33^ DNMT1 has been verified to be an epigenetic inactivation protein that can lead to the downregulation of its targets.^34^, ^35^, ^36^ In this study, we unveiled that DNMT1 could strongly bind PDLIM3 promoter and negatively regulate the expression of PDLIM3 in ecESCs. Functionally, we determined that PDLIM3 knockdown suppressed the proliferation and migration of ecESCs. On the contrary, overexpression of PDLIM3 promoted the proliferation and migration of nESCs. It was proved that high expression of PDLIM3 promoted the progression of Ems.

Dysfunctional immune activation such as macrophage polarization in Ems patients contributes to disease development.^37^ Previous studies have shown an increased number of M2 macrophages in peritoneal fluid and endometrial lesions in patients with Ems.^38^ In this study, we also demonstrated increased macrophage content in diseased tissues. In healthy women without Ems, macrophages have phagocytic properties that allow them to eliminate menstrual debris and maintain homeostasis. Conversely, macrophages grow in Ems lesions because their phagocytic capacity is reduced, promoting angiogenesis and fibrosis, oxidative stress‐induced chronic inflammation, interactions with nerves, estrogen dependence and progesterone resistance.^39^ These findings suggest that M2 macrophages play an important role in the development of endometrial lesions. Ems‐derived exosomes can also affect macrophage polarization.^40^ For example, Wang et al. suggested that fractalkine (FKN) secreted by ecESCs would enhance the production of IL‐10 and inhibit the production of IL‐12, thereby inducing M2 polarization of macrophages, thus facilitating the proliferation and invasion of ESCs.^41^ As reported, PDLIM3 is associated with macrophage M2 polarization in Ems patients.^22^ In our study, it was found that macrophages could capture exosomes derived from ecESCs, and then PDLIM3 was enhanced in macrophages due to direct transfer of PDLIM3 protein via ecESCs‐Exos. Moreover, we uncovered that when PDLIM3 in ecESCs was inhibited or exosome inhibitors were used, the polarization of THP‐1‐DM to M2 macrophages induced by ecESCs coculture was completely reversed. It was demonstrated that ecESCs‐secreted exosomes transfer PDLIM3 to macrophages to induce M2 polarization. Moreover, we discovered that PDLIM3 interacted with NAA40 to suppress its ubiquitination and enhance its level in THP‐1‐DM.

NAA40‐mediated N‐acH4 controls the expression of a specific cohort of genes by regulating the deposition of the adjacent H4R3me2a mark.^29^, ^30^ We found the binding of PDLIM3 and NAA40 in THP‐1‐DM cells by mass spectrometry, and H4R3me2a decreased significantly after interference with NAA40. In human cells, H4R3 is targeted by various protein arginine methyltransferases (PRMT), resulting in different methylation states. Specifically, PRMT1 catalyzes H4R3me2a, PRMT5 deposits symmetric dimethylation to form symmetric dimethylation of H4R3 (H4R3me2s), and PRMT7 mediates H4R3me2s, but primarily monomethylates this histone residue to form H4R3me1.^42^ More studies have shown that the differential expression of PRMT1 is related to the polarization of macrophages.^43^ Therefore, we further studied the relationship between NAA40 and PRMT1, and confirmed that NAA40 binds to the PRMT1 promoter region, mediates the PRMT1 promoter region H4R3me2a, and enhances PRMT1 expression. Moreover, we proved that macrophage M2 polarization is promoted by ecESCs‐Exos via PDLIM3/PRMT1 axis, which in turn contributes to ESCs proliferation and migration. Furthermore, data of animal models also supported that ecESCs‐Exos transfers PDLIM3 to accelerate macrophage M2 polarization to aggravate Ems progression.

## 5 CONCLUSIONS

In conclusion, the present study demonstrated that PDLIM3 was upregulated in ESCs obtained from Ems patients. PDLIM3 promoted the proliferation and migration of both nESCs and ecESCs. PDLIM3 could be delivered by ecESCs‐Exos to macrophage to induce M2 polarization by stabilizing NAA40 to upregulate PRMT1, and such macrophages further contributes to ESCs proliferation and migration, finally aggravating Ems progression.

## AUTHOR CONTRIBUTIONS

Chi Chi and Weizeyu Liu administrated the whole research. Bing Han and Shuzhen Xiang performed the experiments. Jie Chen and Yi Zhang completed the figures. The paper was co‐written by Jie Zhang and Min Li.

## CONFLICT OF INTEREST STATEMENT

The authors declare no conflicts of interest.

## ETHICS STATEMENT

All experiments involving human participants and animals in this study were approved by the Institutional Review Committee of the First Affiliated Hospital of Soochow University. Every participant signed a written informed consent form before any surgical procedure was performed.

## Supporting information

Supporting Information S1

Figure S1

Figure S2

Figure S3

## Data Availability

The data that support the findings of this study are available from the corresponding author upon reasonable request.
