# Supplementary material for: Ectopic endometrial stromal cells‐derived exosomes accelerate endometriosis progression by delivering PDLIM3 to induce macrophage M2 polarization
Source: J Cell Commun Signal. 2026 Oct 23;20(4):e70122. doi: 10.1002/ccs3.70122 (PMC13691930; doi:10.1002/ccs3.70122)
Supplement: Supplementary file 1 [file CCS3-20-e70122-s001.pdf]

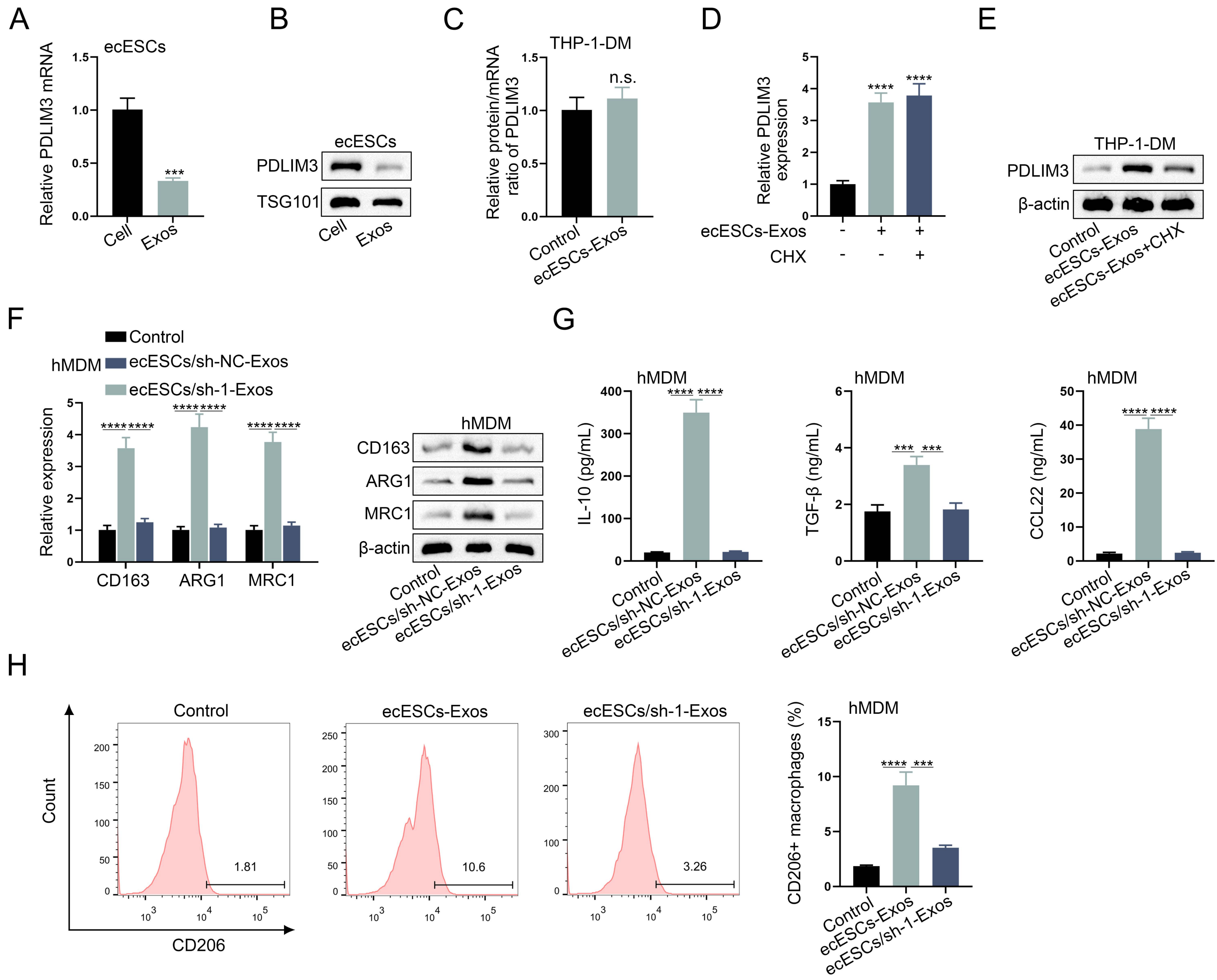
A
ecESCs
Relative PDLIM3 mRNA
Cell
Exos
B
ecESCs
PDLIM3
TSG101
Cell
Exos
C
THP-1-DM
Relative protein/mRNA ratio of PDLIM3
n.s.
Control
ecESCs-Exos
D
Relative PDLIM3 expression
ecESCs-Exos
CHX
E
THP-1-DM
PDLIM3
β-actin
Control
ecESCs-Exos
ecESCs-Exos+CHX
F
Control
hMDM
ecESCs/sh-NC-Exos
ecESCs/sh-1-Exos
Relative expression
CD163
ARG1
MRC1
hMDM
CD163
ARG1
MRC1
β-actin
Control
ecESCs/sh-NC-Exos
ecESCs/sh-1-Exos
G
hMDM
IL-10 (pg/mL)
TGF-β (ng/mL)
CCL22 (ng/mL)
Control
ecESCs/sh-NC-Exos
ecESCs/sh-1-Exos
H
Control
ecESCs-Exos
ecESCs/sh-1-Exos
Count
CD206
1.81
10.6
3.26
hMDM
CD206+ macrophages (%)
Control
ecESCs-Exos
ecESCs/sh-1-Exos
