# Supplementary material for: Ectopic endometrial stromal cells‐derived exosomes accelerate endometriosis progression by delivering PDLIM3 to induce macrophage M2 polarization
Source: J Cell Commun Signal. 2026 Oct 23;20(4):e70122. doi: 10.1002/ccs3.70122 (PMC13691930; doi:10.1002/ccs3.70122)
Supplement: Supplementary file 2 [file CCS3-20-e70122-s003.pdf]

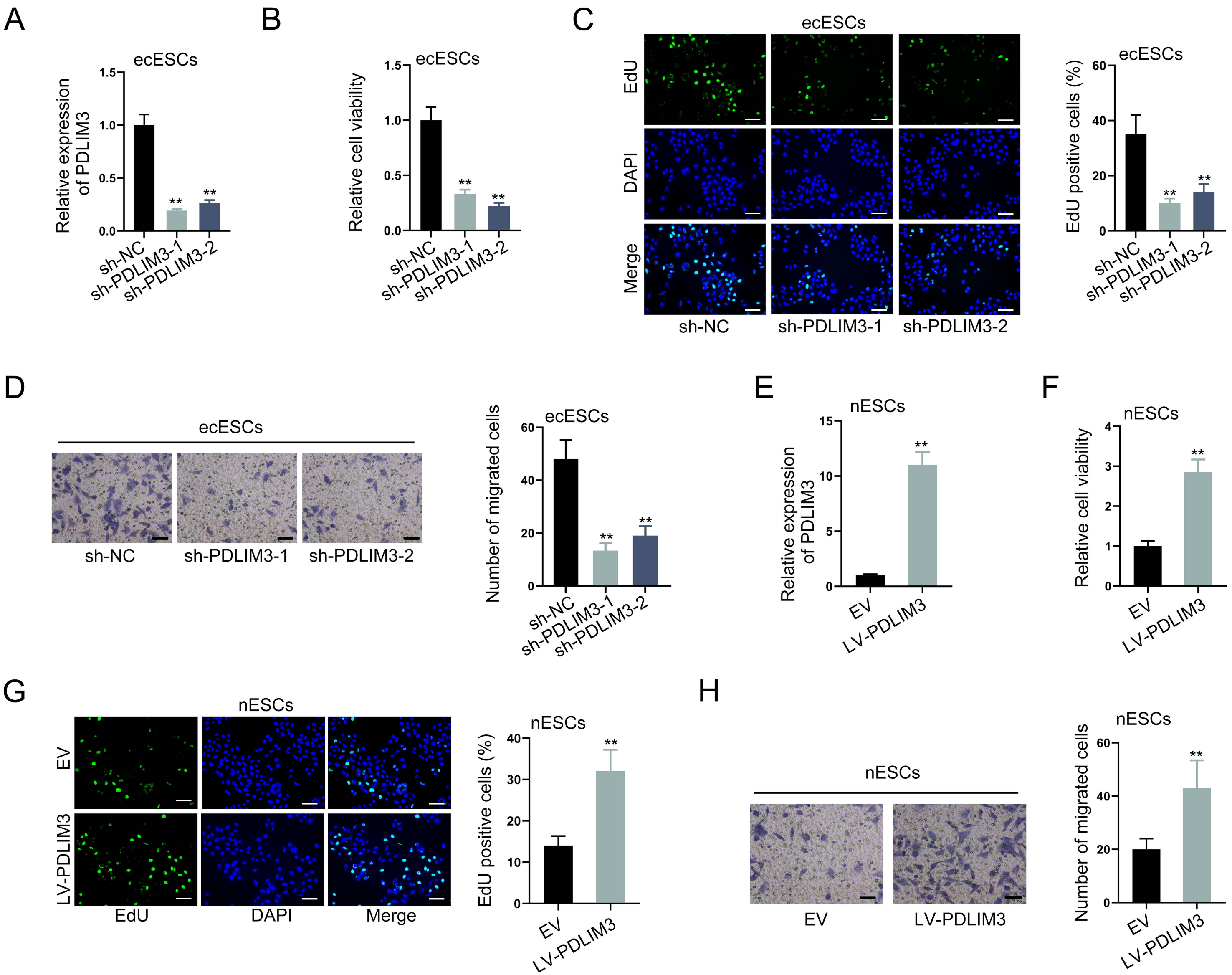

A
ecESCs
Relative expression of PDLIM3
1.5
1.0
0.5
0.0
**
**
sh-NC
sh-PDLIM3-1
sh-PDLIM3-2
B
ecESCs
Relative cell viability
1.5
1.0
0.5
0.0
**
**
sh-NC
sh-PDLIM3-1
sh-PDLIM3-2
C
ecESCs
EdU
DAPI
Merge
sh-NC
sh-PDLIM3-1
sh-PDLIM3-2
ecESCs
EdU positive cells (%)
60
40
20
0
**
**
sh-NC
sh-PDLIM3-1
sh-PDLIM3-2
D
ecESCs
sh-NC
sh-PDLIM3-1
sh-PDLIM3-2
ecESCs
Number of migrated cells
60
40
20
0
**
**
sh-NC
sh-PDLIM3-1
sh-PDLIM3-2
E
nESCs
Relative expression of PDLIM3
15
10
5
0
**
EV
LV-PDLIM3
F
nESCs
Relative cell viability
4
3
2
1
0
**
EV
LV-PDLIM3
G
nESCs
EV
LV-PDLIM3
EdU
DAPI
Merge
nESCs
EdU positive cells (%)
40
30
20
10
0
**
EV
LV-PDLIM3
H
nESCs
EV
LV-PDLIM3
nESCs
Number of migrated cells
60
40
20
0
**
EV
LV-PDLIM3
