# Supplementary material for: Ectopic endometrial stromal cells‐derived exosomes accelerate endometriosis progression by delivering PDLIM3 to induce macrophage M2 polarization
Source: J Cell Commun Signal. 2026 Oct 23;20(4):e70122. doi: 10.1002/ccs3.70122 (PMC13691930; doi:10.1002/ccs3.70122)
Supplement: Supplementary file 3 [file CCS3-20-e70122-s004.pdf]

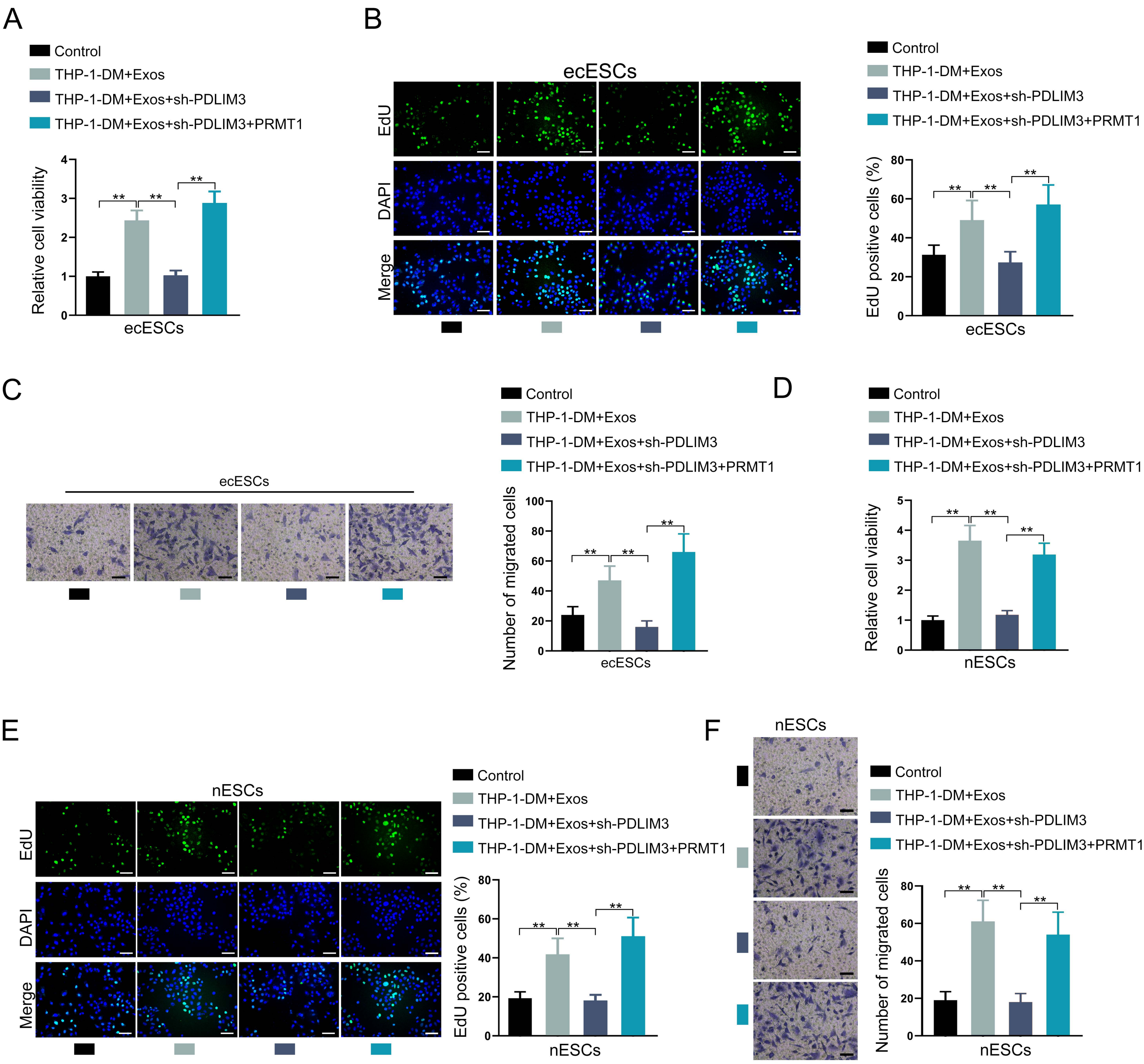

A
Control
THP-1-DM+Exos
THP-1-DM+Exos+sh-PDLIM3
THP-1-DM+Exos+sh-PDLIM3+PRMT1
Relative cell viability
ecESCs
B
ecESCs
EdU
DAPI
Merge
EdU positive cells (%)
ecESCs
C
ecESCs
Number of migrated cells
ecESCs
D
Relative cell viability
nESCs
E
nESCs
EdU
DAPI
Merge
EdU positive cells (%)
nESCs
F
nESCs
Number of migrated cells
nESCs
