# Supplementary material for: Ectopic endometrial stromal cells‐derived exosomes accelerate endometriosis progression by delivering PDLIM3 to induce macrophage M2 polarization
Source: J Cell Commun Signal. 2026 Oct 23;20(4):e70122. doi: 10.1002/ccs3.70122 (PMC13691930; doi:10.1002/ccs3.70122)
Supplement: Supplementary file 4 [file CCS3-20-e70122-s002.docx]

**Figure S1 PDLIM3 promotes proliferation and migration of ESCs**

**A.** PDLIM3 was knocked down in ecESCs with specific shRNAs. **B.** The viability of ecESCs with or without PDLIM3 silencing was assessed by CCK-8 assay. **C.** The proliferation of ecESCs with or without PDLIM3 silencing was assessed by EdU assay. **D.** The migrating ability of ecESCs was detected by transwell assay before or after knockdown of PDLIM3. **E.** RT-qPCR proved that PDLIM3 expression was overexpressed in nESCs. **F.** The viability of nESCs with or without PDLIM3 overexpression was assessed by CCK-8 assay. **G.** The proliferation of nESCs with or without PDLIM3 overexpression was detected by EdU assay. **H.** The migrating ability of nESCs with or without PDLIM3 overexpression was measured by transwell assay. ^**^P<0.01.

**Figure S2 Exosomes from ecESCs directly transfers PDLIM3 protein to facilitate macrophage M2 polarization**

**A-B.** RT-qPCR and western blot validated the existing of both PDLIM3 mRNA and protein in ecESCs-Exos. **C.** The ratio of PDLIM3 protein/mRNA was quantified in THP-1-DM with or without ecESCs-Exos treatment. **D-E.** RT-qPCR and western blot tested the changes in PDLIM3 mRNA and protein levels in THP-1-DM treated with ecESCs-Exos or further addition of CHX. **F.** RT-qPCR and western blot assessed the expression of M2 markers in hMDM before or after treating with exosomes from control or PDLIM3-silenced ecESCs. **G.** The secretion of several cytokines in THP-1-DM cells under different treatments were examined via ELISA. **H.** Flow cytometry analyzed the percent of CD206-positive macrophages in indicated THP-1-DM cells. ^***^P<0.001, ^****^P<0.0001; n.s.=not significant.

**Figure S3 Exosomal PDLIM3 promotes ESCs growth and migration through upregulating PRMT1 to induce macrophage M2 polarization**

**A-B.** The viability and proliferation of ecESCs treated with different exosomes or plasmids were detected by CCK-8 and EdU assays. **C.** The migration of ecESCs was evaluated by transwell assay after being treated with different exosomes or plasmids. **D-E.** The viability and proliferation of nESCs treated with different exosomes or plasmids were detected by CCK-8 and EdU assays. **F.** The migration of nESCs was evaluated by transwell assay after being treated with different exosomes or plasmids. ^**^P<0.01.
